# Adenomata of the Pharynx, with Report of Cases

**Published:** 1891-03

**Authors:** L. M. Crichton

**Affiliations:** Atlanta, Ga.


THE ATLANTA MEDICAL AND SURGICAL JOURNAL.

VOL. VIII. MARCH, 1891. NO. 1.

### Original Communications.

### ADENOMATA OF THE PHARYNX, WITH REPORT OF CASES.

BY L. M. CRICHTON, M. D., ATLANTA, GA.

"There is nothing new under the sun." Especially is this true as regards adenoids. The brilliant and thorough work of Wilhelm Meyer left but little to be learned by his successors.

In this article I wish to interest the general practitioner in a disease which has not received the attention from him that its great practical importance demands; to give him in a condensed form what is known of the disease, how it is recognized, how treated, and report some cases. Because a man is not a specialist, it is no reason or excuse for his being unable to diagnose at least the simpler forms of disease of special parts.

Many practitioners never interrogate the nose and throat to find out if there is a condition there causing the anæmia, or insomnia, or depressed spirits, or asthma, etc., etc., which they have been treating with but little benefit. That such conditions do exist and do affect the general health profoundly, is absolutely beyond question.

SYNONYMS: Adenoids, adenoid growths, adenoid vegetations, hypertrophic posterior nasal pharyngitis, hypertrophy of pharyngeal tonsil, adenomata of pharynx.

The recognition of hypertrophy of the tissues at the vault of the pharynx dates back to the time of William Hunter. In 1862, "Naso-palatine Gland Disease" appeared from the pen of Sir Andrew Clark. Löwenberg and Volotolini wrote of aural complications, in 1865.

The subject was left, however, to be exhaustingly studied both from a clinical and atomical point of view, by Wilhelm Meyer, of Copenhagen, in 1868. So thoroughly did he investigate the 102 cases on whom he operated, that little of much value has been added to his work.

Hypertrophy of the pharyngeal tonsil is essentially a disease of early life, only a very small percentage occurring after the thirtieth year.

Of Meyer's 102 cases seventy-two were under fifteen years; twenty-one were between fifteen and twenty years; eleven cases were between twenty and twenty-five years.

Of Bosworth's seventy-five cases twenty-one were under fifteen years; twenty-seven between fifteen and twenty; twenty-three between twenty and thirty.

Bosworth accounts for the difference in the average age of his and Meyer's cases by saying: "In many instances the small, glandular hypertrophies found in adult life are identical with those of child life, and are therefore to be treated on the same principles," and "that he has included in his tables a *large number* (italics mine) of those broad, flat growths which constitute the essential condition of many cases of so-called naso-pharyngeal catarrh." Including these cases will of course make the average age greater.

The etiology of adenoids has not been positively settled, the authorities differing. Löwenberg believes the lymphatic temperament to be the "cause of the disease in the very large majority of cases he has seen." Sajous, "probably traceable to a catarrhal state of the naso-pharynx."

Lennox Browne, citing Hill, agrees with him and associates insanitary surroundings and adenoids, and states: "Lymphatic temperament predisposes to septic inflammation, which may in time be followed by further hypertrophy.

Bosworth, "simply a tendency to hypertrophy under stimulus of repeated colds." Although none of these theories are thoroughly satisfactory, I like that of Bosworth's better than the others.

The various symptoms of adenoids are of course not all manifest even in a typical case. I will give the symptoms in their order of importance: audition, deafness, impaired hearing, otitis media catarrhalis chronica, otitis media purulenta chronica.

The symptom or complication of the ear cannot be too forcibly dwelt upon when we think of the vast importance of this special sense and the ease with which the trouble is prevented, stopped in its course, or ameliorated by a prompt and ready recognition of the cause and the efficient removal of the same. The extreme simplicity of the diagnosis places it in the hands of every practitioner to recognize the trouble, and there can be no possible excuse for a physician who allows a child to become deaf from adenoids, *provided* he has seen the case before very serious impairment of hearing has begun.

Bosworth says, "probably a large majority of ear trouble in children, under the age of twelve years, is dependent upon vegetation at the vault of the pharynx."

Woakes gives ninety-five per cent. of aural complication.

Meyer, in 102 cases, gives aural complications in seventy-two.

Urbantschitsch, in 175 cases, gives aural complications in 130.

Swinburne, in forty-two cases, gives aural complications in twenty-seven.

Bosworth, in seventy-five cases, gives aural complications in twenty-eight.

Respiration: Patient breathes with mouth open. The amount of mouth breathing is in proportion to the extent to which the adenoids have encroached upon the post-nasal space. In some cases it occurs only during sleep; in others, during the day the mouth is only partly open. In still other cases, breathing takes place through the mouth both day and night. Adenoid patients, as a rule, snore during sleep, and wake in the morning with the throat and mouth dry.

In many instances the child will be brought to you for a "catarrh," nothing else being complained of. You will find in the nose a mucous, muco-purulent or purulent grayish discharge, with masses of desicated mucus and pus. When you see such a condition in a child *never fail to interrogate the vault of the pharynx.* In pronounced cases the nose is broadened and flattened at its base.

Voice production: Voice has a muffled character; want of resonance; patient "talks thick;" deadness of voice; pronounces m, "eb," and n, "ed." Child is likely to sleep poorly; restless in sleep; may have headaches, disordered digestion, etc., etc.

These adenoid patients, as a rule, seem backward, dull and stupid (varying with amount of naso-pharyngeal trouble); the face has a "blank look," sometimes almost idiotic. This condition comes probably, as Bosworth suggests, from the impaired hearing rather than from mental deterioration.

In a certain number of cases, Fränkel, Bosworth, Chattelier, reflex asthma has been caused by these growths.

Lennox Browne dwells very forcibly on the relation of adenoids to false croup. He says: "In these cases reflex croup and cough are not infrequent; indeed, I believe that in almost all, if not all cases of laryngismus stridulus or false croup, the subjects would, if examined, be found to be mouth-breathers."

He also holds that adenoids predispose to "attacks of diphtheria, of exanthemata and other fevers exhibiting nasal and pharyngeal inflammations."

Diagnosis: With one or more of the above symptoms to direct our attention to the naso-pharynx, a diagnosis is a very simple matter.

The most positive and easiest way to verify a diagnosis from symptoms, and to place its accuracy beyond question, is digital examination. Bosworth objects to examination with the finger and advises the employment of the rhinoscopic mirror. I can see no good reason for his objection. If the digital examination be made *carefully*, there is no danger of doing harm. To the mirror there are the most decided objections, in that general practitioners, as a rule, are not skilled in the use of the mirror; in many children the examination is impossible, even by a skilled hand, on account of the struggles, gags, etc., of the child. Most children, if you have gained their confidence and are not too abrupt, will allow the finger to be inserted, for the first time, into the naso-pharynx with but little resistance.

Standing to the left side and somewhat behind patient, with right hand press the head lightly against your body, then introduce the index finger of the left hand through the mouth to vault of pharynx and examine it gently. If there be adenoids, they will convey to the bulb of the finger a sensation somewhat similar to that conveyed by the rugæ of the vaginal walls of a nulliparous woman, or, to use a classical comparison, "like a mass of earthworms." Press the finger lightly in direction of the vault; remove the finger, and its tip will be stained with blood. Your diagnosis is now positive, and if your manipulations have been careful, no harm has been done. This is the fimbriated variety.

The second variety is a cushion-like mass with deep, depressions irregularly in its surface, and conveys a corresponding impression to the finger.

Treatment: Radical surgical measures are here most plainly and positively indicated and are fully warranted.

Treatment by medication, as astringent sprays, applications, etc., are not indicated and produce only "a modification of the symptoms. A cure *can* be effected by means of chemical or potential cautery, but it takes many applications and a long course of treatment, and seems to me a very roundabout way to accomplish so simple a result. A number of operations have been devised for the removal of these growths, and every operator of any great repute has invented an instrument or modified one already in use to suit himself.

After a careful study of the different operations and instruments, I believe the means are not of such vast importance, although some are, of course, preferable to others. It *is* important, however, that the surgeon should be *careful*, know his anatomy, exactly what he intends to do, and that he does it with reasonable skill.

Lennox Browne scraps these growths from the vault of the pharynx with his finger nail. Sajous employs the galvano-cautery snare ; Meyer, a ring knife ; Bosworth, the cold wire snare. Löwenburg, Curtis, Cohen, Schesch and others use the post-nasal forceps.

The question of anæsthesia is one on which the authorities are divided. Some employ an anæsthetic, others do not. The surgeon must be governed by the case and circumstances, and decide the matter for himself. Anæsthetics are not *absolutely free* from danger ; the operation is short and not very painful.

In my own work, if the patient or parents of patient are tractable and willing to submit to some pain, I prefer operating without anæsthesia. If compelled to use an anæsthetic, I would prefer nitrous oxide gas, as used by Lennox Browne. Prognosis is always good ; all the symptoms are relieved. As to audition, if the hearing be much impaired its progress will be stopped and the hearing probably improve. If but slightly impaired, the prognosis for recovery is good. If the ear be not affected at the time of diagnosis of adenoids, the operation, as a prophylactic measure, is indicated and thoroughly justifiable.

CASES.

Annette N., age 4 years, kindly referred to me by Dr. McRae. Mother a strong, vigorous woman. Mother said she had noticed an offensive odor to breath, more at one time than at another, and a discharge from nose. Said child slept with mouth open, and kept mouth partly open during the day. Douche had been used with only temporary benefit. Child thin and delicate looking. Face had a " pinched look." Mouth partly open, giving the face a blank expression. Hearing not affected.

On examination of nose, found a greyish discharge and desiccated mucus on septum and turbinateds. On account of age, etc., of child, a post-rhinoscopic examination with mirror was not practicable, so I made the examination with my index finger. This made positive what I had surmised from history, symptoms and appearance of child—adenoid growths. They were of the fimbriated variety, and almost entirely filled the vault. I explained the condition to the mother, and advised operation, telling her it would cure the catarrh and improve the general health of the child ; she consented to operation.

Put patient on spray of Sol. Dobell, diluted one-half, to get nose clean. One week later, Drs. McRae and Giddings being present, I removed a mass of adenoid tissue from the vault of the pharynx. My method of operating is as follows : Child is held firmly on lap of assistant, arms of patient pinioned by arms of assistant ; gag placed between teeth on right side, and held, as well as the head, by an assistant behind child. Standing in front, I introduce finger of left hand into rhino-pharynx, press uvula and palate up and out of danger. I then introduce post-nasal forceps, closed, along my finger to vault, open them, grasp adenoids, partly cut and partly twist them off. I usually go in five or six times, removing as much as possible. I then introduce my index finger and scrape off what remains.

Considerable blood is lost during the removal of the growths, but the hemorrhage is never serious in the fimbriated variety, ceasing after a few minutes of its own accord.

Although free from danger, four things must, when forceps are used, be carefully kept in mind. The uvula must be kept from between the blades of forceps, else it will be lacerated and crushed. The posterior border of the septum must not be grasped and cut in place of the adenoids. Great care must be taken not to injure the openings of the Eustachian tubes. The mucous membrane must not be stripped from the walls of the rhino-pharynx.

After operation told mother to continue spray. Two weeks from operation reported entire relief from all symptoms. Ten days later, the mother puzzled me not a little by reporting a return of the unpleasant odor to breath. I examined the nose and found a grayish mass in the floor of left nostril, right nostril normal. Decided it was foreign body. It proved to be a piece of cotton which in some way the child had introduced. It was removed and the breath remained free from odor. Three months after operation there had been no return of any symptom. Child had *improved very much in general health.*

I have reported this case somewhat at length, because it belongs to that class of cases in which a general practitioner is apt to look in the *nose* for the trouble.

The two following cases are very similar to the one above, so I will report them briefly.

A. G., boy, age, eight years. Family history good. Father, a strong German. Said child's sleep was much disturbed. Slept with mouth open and snored. Discharge from nose. Hearing somewhat impaired. Examination. Boy in a very fair general condition. Mouth partly open. Peculiar facial expression, not very marked. Introduced finger into rhino-pharynx and found adenoid vegetations, fimbriated variety. Advised operation. Operated; making two sittings.

Subsequent history showed entire relief from symptoms.

M. D., girl, age, six years. Family history good. Father vigorous. Symptoms almost exactly same as above, with the exception that the child's hearing was more profoundly affected. Examination revealed adenoids. Advised operation. Operated.

Subsequent history showed disappearance of all symptoms, save the impaired hearing; this, however, was improving when case was last seen.

If this article draws the attention of the practitioner to this important class of cases, and aids him in the investigation of the same, it will have served its purpose, if not "*satis verborum.*"

101 *Whitehall St.*

